# Microbiota of healthy dogs demonstrate a significant decrease in richness and changes in specific bacterial groups in response to supplementation with resistant starch, but not psyllium or methylcellulose, in a randomized cross-over trial

**DOI:** 10.1099/acmi.0.000774.v4

**Published:** 2024-05-14

**Authors:** Silke Salavati Schmitz, Jorge Perez-Accino Salgado, Laura Glendinning

**Affiliations:** 1 Hospital for Small Animals, Royal (Dick) School of Veterinary Studies, College of Medicine and Veterinary Medicine,, University of Edinburgh, Easter Bush Campus, Midlothian, EH25 9RG, UK; 2 The Roslin Institute, University of Edinburgh, Easter Bush Campus, Midlothian, EH25 9RG, UK

**Keywords:** dietary fibre, gastrointestinal, microbiome, prebiotics, canine

## Abstract

Even though dietary fibres are often used as prebiotic supplements in dogs, the effect of individual types of fibres on canine microbiota composition is unknown. The objective of this study was to assess changes in faecal microbiota richness, diversity and taxonomic abundance with three different fibre supplements in dogs. These were psyllium husk, resistant starch from banana flour and methylcellulose. They were administered to 17 healthy dogs in a cross-over trial after transition to the same complete feed. Faecal scores and clinical activity indices were recorded, and faecal samples were collected before and at the end of supplementation, as well as 2 weeks after each supplement (washout). Illumina NovaSeq paired-end 16S rRNA gene sequencing was performed on all samples. After quality control and chimera removal, alpha diversity indices were calculated with QIIME. Differences in specific taxa between groups were identified using Metastats. Methylcellulose significantly increased faecal scores but had no effect on microbiota. Psyllium resulted in minor changes in the abundance of specific taxa, but with questionable biological significance. Resistant starch reduced microbiota richness and resulted in the most abundant changes in taxa, mostly a reduction in short-chain fatty acid-producing genera of the phylum *Bacillota*, with an increase in genera within the *Bacteroidota*, *Pseudomonadota*, *Actinomycetota* and *Saccharibacteria*. In conclusion, while psyllium and methylcellulose led to few changes in the microbiota composition, the taxonomic changes seen with resistant starch may indicate a less favourable composition. Based on this, the type of resistant starch used here cannot be recommended as a prebiotic in dogs.

## Graphical abstract

Microbiota of healthy dogs demonstrate significant changes in specific bacterial groups in response to supplementation with resistant starch (but not psyllium or methylcellulose) in this randomized cross-over trial.

## Data Summary

The paired-read fastq data that support the findings of this study have been deposited in the European Nucleotide Archive with the accession code PRJEB67805 (https://www.ebi.ac.uk/ena/browser/view/PRJEB67805). For the purpose of open access, the authors have applied a Creative Commons Attribution (CC BY) licence to any Author Accepted Manuscript version arising from this submission.

## Introduction

A number of health benefits are associated with the consumption of dietary fibre (DF), including compositional as well as functional changes of the intestinal microbiota (IM); for example plant- and carbohydrate-rich diets result in increased IM richness and bacterial short chain fatty acid (SCFA) production [1].

A low intake of DF does not only lead to reduced IM diversity, but also shifts the gut microbial metabolism away from SCFAs towards less favourable bacterial metabolites, often derived from amino acids (e.g. branched-chain fatty acids, ammonia, phenolic and indolic compounds, and hydrogen sulphide), which can be detrimental to host health [1]. The cytotoxic and pro-inflammatory nature of these metabolites contributes to the development of chronic diseases, particularly an increased prevalence of inflammatory bowel disease (IBD) and colorectal cancer [2].

A number of DFs are also considered prebiotics, defined as ‘a substrate that is selectively utilized by host micro-organisms conferring a health benefit’ [3]. DFs are additionally classified by their physicochemical properties (water solubility and viscosity), as well as their fermentability. In most cases, soluble fibre types are fermented for example into SCFAs more quickly than insoluble types [1].

Chemically, DFs can be divided into three main classes: (1) non-starch polysaccharides (NSPs), which includes non-fermentable fibres such as psyllium seed husk (*Plantago ovata*) (PSY), (2) resistant (non-digestible) oligosaccharides (e.g. fructo-oligosaccharides [4]) and (3) resistant starch (RS), for example granular starches from green bananas [5].

There is evidence that supplementation with specific DF restores some of the health benefits they can infer. Different types of RS [6], cellulose [7] and PSY have specific effects on the IM. For example, RS has been shown to enrich *Bifidobacteria*, *Ruminococcus* and other beneficial bacteria [1]. PSY supplementation induces distinct IM community changes, including higher relative abundance of *Bacteroides* and *Parabacteroides* (fibre-digesting bacterial groups) up to 70 % of the total IM bacteria, with a matching increase in SCFA production, and reduction of *Enterobacteriaceae* and *Pseudomonadaceae* [8]. Even the non-fermentable fibre methylcellulose (MTC) has been shown to modulate IM composition and diversity as well as faecal bile acid metabolism and prevent weight gain in mice fed a high-fat diet [9].

Not only do companion animals such as dogs have a similar IM composition and richness to people [10,11], as they have co-evolved to be able to digest similar food [12] and can hence serve as appropriate models for IM-related interventions, in ‘industrialized’ populations, they also suffer from similar emerging diseases, including chronic inflammatory gut conditions like IBD [11,13]. Different DFs (especially PSY) are commonly used in canine feed or given as supplements to individual pet dogs for a variety of spontaneously occurring intestinal conditions (e.g. ‘fibre-responsive’ colitis [14], irritable bowel syndrome [15]), but very little is known about the effect of specific DFs on dogs’ IM composition or function. One study showed that PSY results in a significant increase of the SCFAs propionate and n-butyrate in faecal samples of dogs after 15 days of supplementation [16]. In addition, PSY might protect against colitis via activation of bile acid receptors in intestinal epithelial cells [17], which could be particularly relevant, as severely altered faecal bile acid metabolism has recently been identified as a hallmark of canine IBD [18].

The goal of this study was to investigate the effect of three commonly used DF types on the IM composition and richness of healthy dogs to assess their potential health benefits and create an evidence-base for their use as prebiotics in both dogs and potentially people.

## Methods

### Animals, feeding interventions and clinical scores

**Fig. 1.**
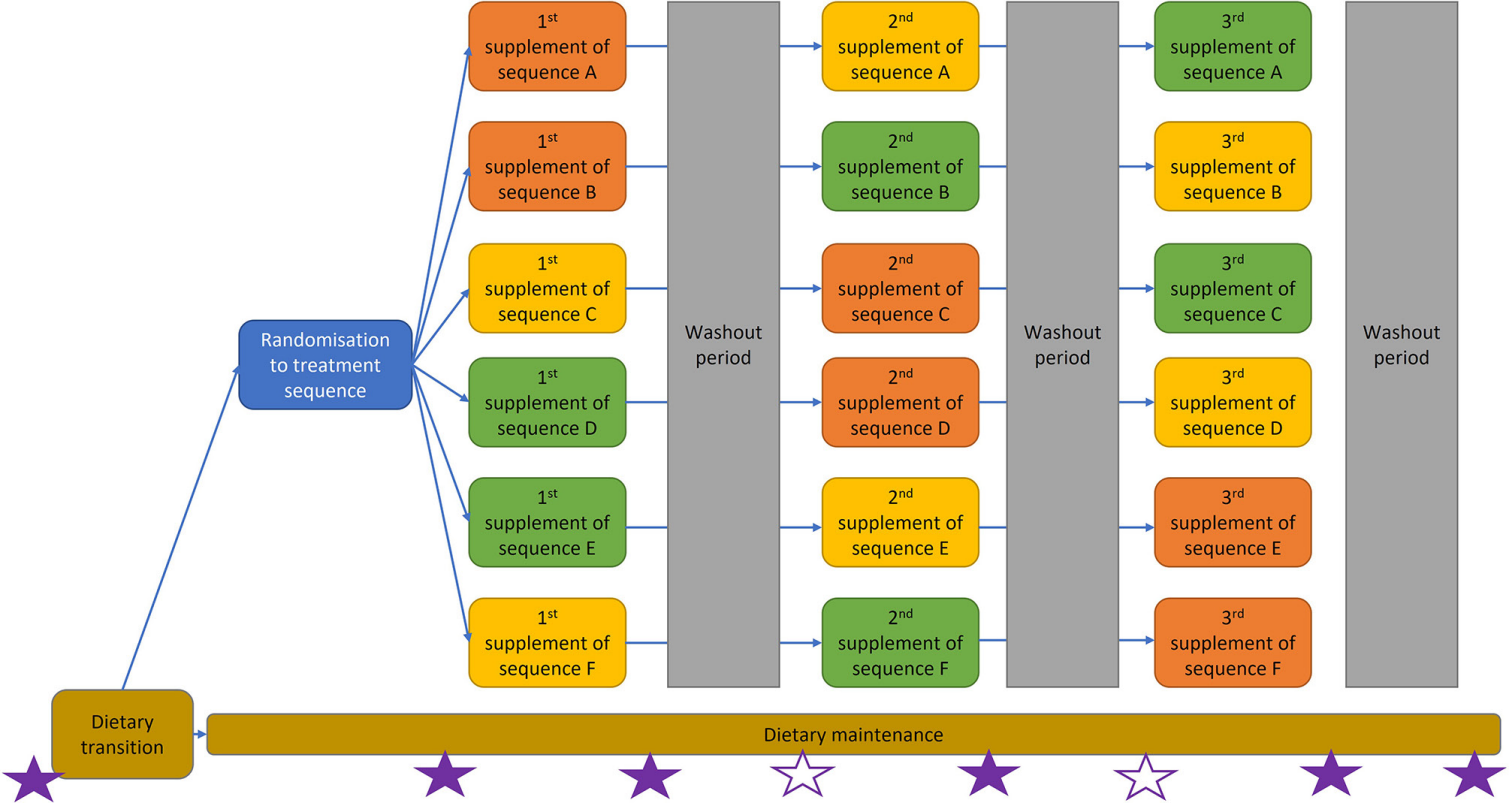
Illustration of the design and analysis of the crossover fibre supplementation trial. Six separate supplementation sequences were designed (A–F). Different coloured supplement boxes represent the three different dietary fibres used. Filled stars indicate time points for faecal sampling and owner questionnaires; open stars indicate time points for faecal sampling only.

Dogs recruited for the study were privately owned pets and deemed healthy based on the absence of clinical signs and normal physical examination findings (Table S1, available in the online version of this article). They all had to be regularly wormed and treated for ectoparasites, and not have travelled abroad. Dogs on a prescription diet or any regular medication or supplements were excluded. Owners were asked to collect a freshly voided faecal sample (day −14) before transitioning all dogs to the same commercial complete dry dog food (Hill’s Science Plan Advanced Fitness medium adult tuna and rice) at 1.4–1.8 Resting Energy Requirements (RER) depending on lifestyle for 2 weeks, after which another faecal sample was collected (day 0, baseline). After that, dogs were maintained on the same diet and randomized to receive each of the three study fibre supplements for a duration of 2 weeks, followed by a 2 week washout period before the next supplement (Fig. 1). Throughout the duration of the study, the dogs’ diet was not permitted to be changed. Fresh faecal samples were collected on the last day of each supplementation and washout period (days 14, 28, 42, 56, 70 and 84). Owners were asked to keep a diary that included daily confirmation of supplement administration (which were labelled A, B and C in otherwise plain dispensing bags), and scoring of each naturally voided faecal sample’s consistency using a pictorial template of a well-established tool (Purina Faecal Score; PFS), where score 1 represents very hard and dry faeces to score 7, which represents watery faeces with no texture. In addition, owners were asked to fill in a validated questionnaire, the Canine IBD Activity Index (CIBDAI [19]) at day −14 and 0, on the last day of each supplement (days 14, 42 and 70) and after a final washout of 2 weeks (end of study, day 84).

DF supplements used were commercially available food-grade additives, namely PSY husk (Colon Care Plus; Holland and Barrett), a resistant starch (Green Banana Flour; Natural Evolution) and MTC (Methocel; SpecialIngredients). Dosing was identical for all three DFs, twice daily and based on an individual dog’s body weight: dogs <5 kg received 2 g, dogs 5–10 kg 4 g, dogs >10–30 kg 8 g, dogs >30–50 kg 12 g and dogs >50 kg 16 g of each supplement. This dosage was derived from empirically available doses for PSY [20]. Supplements were advised to be given with the normal food ration and using a specific measuring spoon provided with specific dosing instructions for each supplement and dog to allow administration of the correct amount.

### Faecal DNA extraction, 16S rRNA gene amplification and sequencing

Faecal samples were aliquoted into sterile 5 ml Bijoux tubes within 60 min of receipt at the hospital and stored at −80 °C until the time of analysis. DNA extraction was performed as described previously [21] using the DNeasy PowerLyzer PowerSoil Kit (Qiagen).

Extracted genomic DNA was sent to a commercial service provider (Novogene; www.novogene.com) for 16S rRNA gene PCR amplification, DNA sample quality control, amplicon library preparation and Illumina NovaSeq paired-end sequencing with 30× coverage. Briefly, DNA concentration and purity was assessed on 1 % agarose gels and DNA was diluted to 1 ng ml^−1^. The V4 region of the 16S rRNA gene was amplified using the primers GTGCCAGCMGCCGCGGTAA and GGACTACHVGGGTWTCTAAT with barcodes. All PCRs were carried out with Phusion High-Fidelity PCR Master Mix (New England Biolabs), resulting in an amplicon of ~300 bp in size. PCR products were mixed at equal density ratios, purified with a Qiagen Gel Extraction kit, and the library generated with NEBNext Ultra DNA Library Prep kit for Illumina (quantified via Qubit and quantitative PCR).

### Sequence data processing, OTU clustering, taxonomic annotation and diversity analysis

Primer sequences were removed, then paired-end reads were merged using FLASH (v.1.2.7) [22]. Quality filtering was used to obtain high-quality clean reads using the split_libraries_fastq.py command from QIIME (v.1.7.0) [23,24]. Chimeras were detected using UCHIME [25] with the silva reference database (v.138.1) [26], then removed. Operational taxonomic unit (OTU) clustering at ≥97 % similarity was performed using UPARSE (v.7.0.1090). A representative sequence for each OTU underwent taxonomic assignment, using the QIIME (v.1.7.0) command assign_taxonomy.py (mothur method) with the silva database [26].

### Statistical analysis

OTU counts were normalized by subsampling to the sample with the fewest counts (39 442 reads). Relative abundance values are reported throughout as a value between 0 and 1, where 0 indicates the complete absence of the taxa and 1 indicates that the taxon is 100 % abundant. Subsequent alpha and beta diversity analyses were all performed on these normalized counts. Alpha diversity indices, including observed-species (OTUs), Chao1 and Shannon, were calculated with QIIME (v.1.7.0). Significant differences in specific taxa between groups were identified using Metastats [27], with the Benjamini and Hochberg false discovery rate [27] used for correcting for multiple tests (adjusted *P*-value=*q*-value). Non-metric multidimensional scaling (NMDS) graphs were constructed using values produced by metaMDS from the vegan (v.2.6.4) package, using Bray–Curtis dissimilarity values. Permutational multivariate analyses of variance (PERMANOVAs) were conducted using Bray–Curtis dissimilarity values, and the adonis2 command from the vegan (v.2.6.4) package. The significance of differences in abundance between groups of specific genera of interest was calculated using the Wilcoxon test.

Clinical data (CIBDAI and PFS) as well as alpha diversity indices were analysed and compared using GraphPad Prism 9.5.1 for Windows (GraphPad Software; www.graphpad.com). Data were tested for normality using Shapiro–Wilk tests and compared using Kruskall–Wallis tests with Dunn’s multiple comparison test as post-hoc analysis.

## Results

### Animal characteristics

A total of 24 dogs were initially recruited. Of those, five did not reach the end of their first supplementation stage due to palatability issues with the supplement. Two further dogs were removed from the study as they developed unrelated medical problems that prevented them from completing the study. Data from the remaining 17 dogs were included in the analysis. However, from two of those dogs, no faecal sample was collected at day 0 as they were accidentally transitioned to the first supplement too early. Another two dogs completed the first and second supplementation and washout phases but developed diarrhoea with the third supplement (MTC), so they were taken off this supplement and a final sample was collected at day 84. Hence, a complete dataset was available for 13/17 dogs.

For the 17 dogs, the order of DF supplements given can be found in Table S2 and the available samples in Table S3.

### MTC, but not PSY or RS, causes an increase in faecal scores, which returned to normal at the end of washout periods

**Fig. 2.**
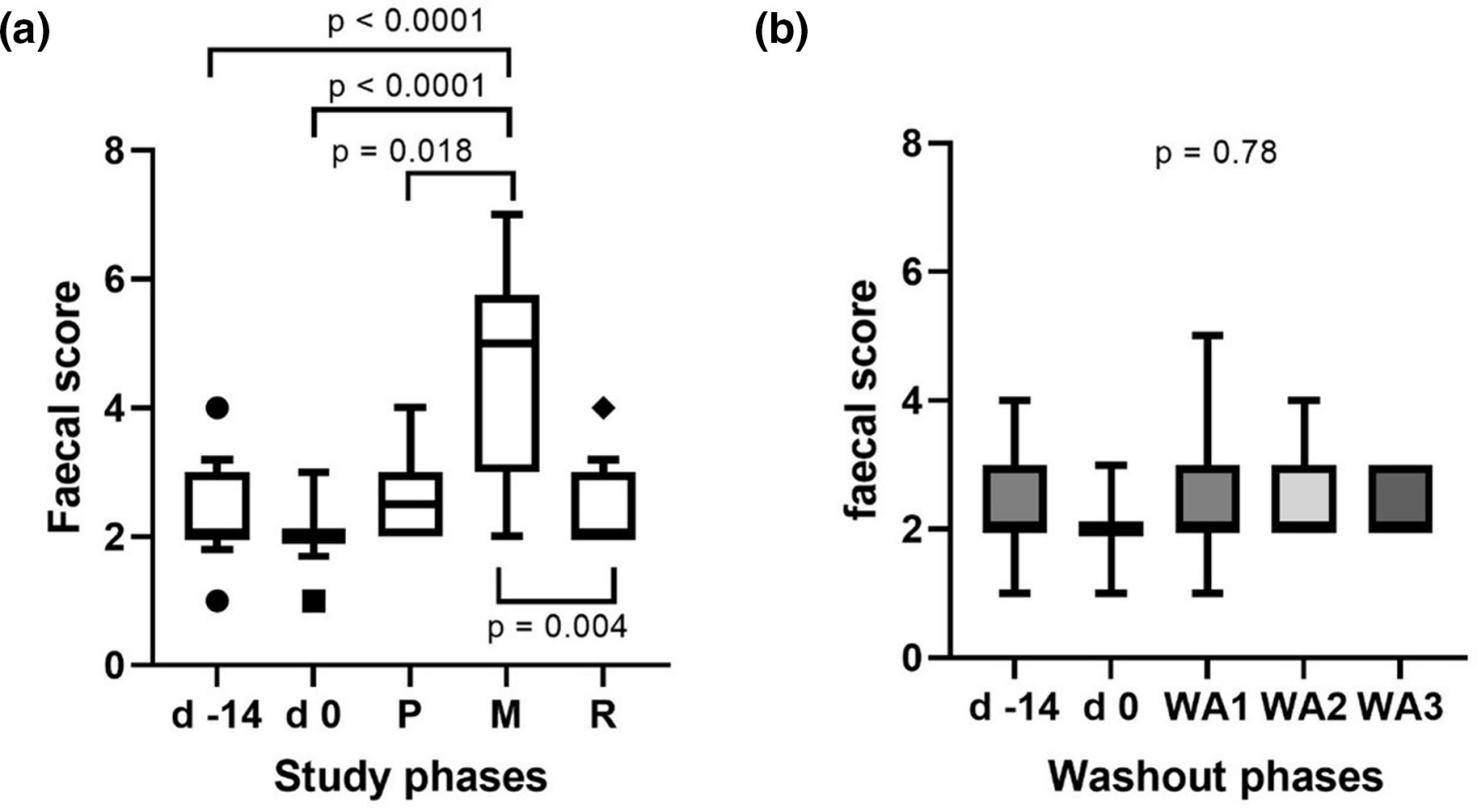
Faecal scores throughout the different supplementation phases (**a**) and at the end of each washout (WA) phase (**b**) for 17 dogs participating in the study. The higher the score the softer the stools: d −14=before change to a standardized diet at day 0;, P=psyllium husk, M=methylcellulose, R=resistant starch, WA=washout phase.

Faecal scores at the start of the study were a median of 2 (range 1–4) and did not significantly change with the dietary transition (median of 2, range 1–3) (*P*>0.99). There was also no difference between baseline samples and supplements with the exception of MTC, resulting in a median PFS of 5 (range 3–7) (Fig. 2a); and there was no significant change in faecal scores between the different washout periods (*P*=0.7; Fig. 2b).

**Fig. 3.**
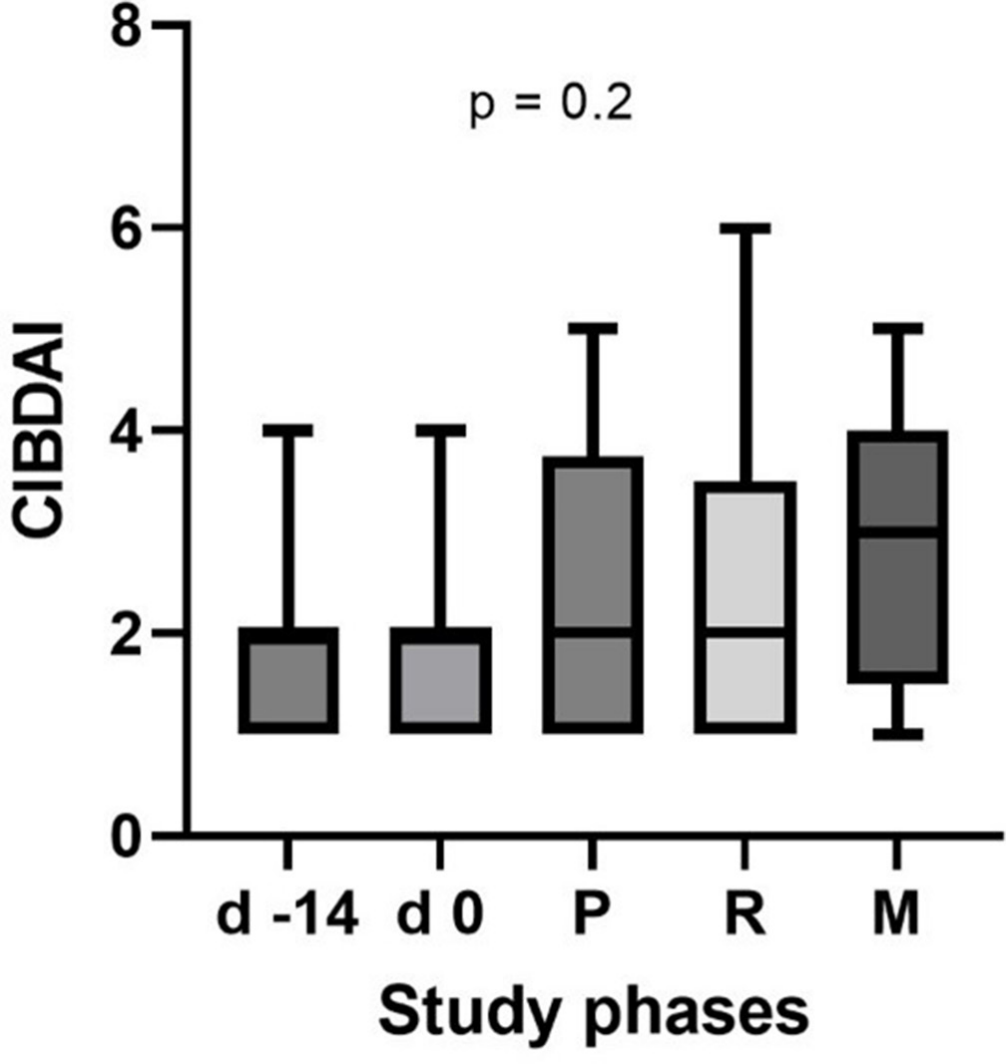
Canine inflammatory bowel disease activity index (CIBDAI) for the 17 dogs included in the feeding of different DF supplements. d −14=before diet change, d 0=after diet change, P=psyllium husk, R=resistant starch, M=methylcellulose.

CIBDAI values were low at the start (day −14) with a median of 2 (range 1–4), and remained low throughout the supplementation periods, with no significant differences between DF supplements (see Fig. 3).

### Dietary change did not significantly influence the intestinal microbiota

**Fig. 4.**
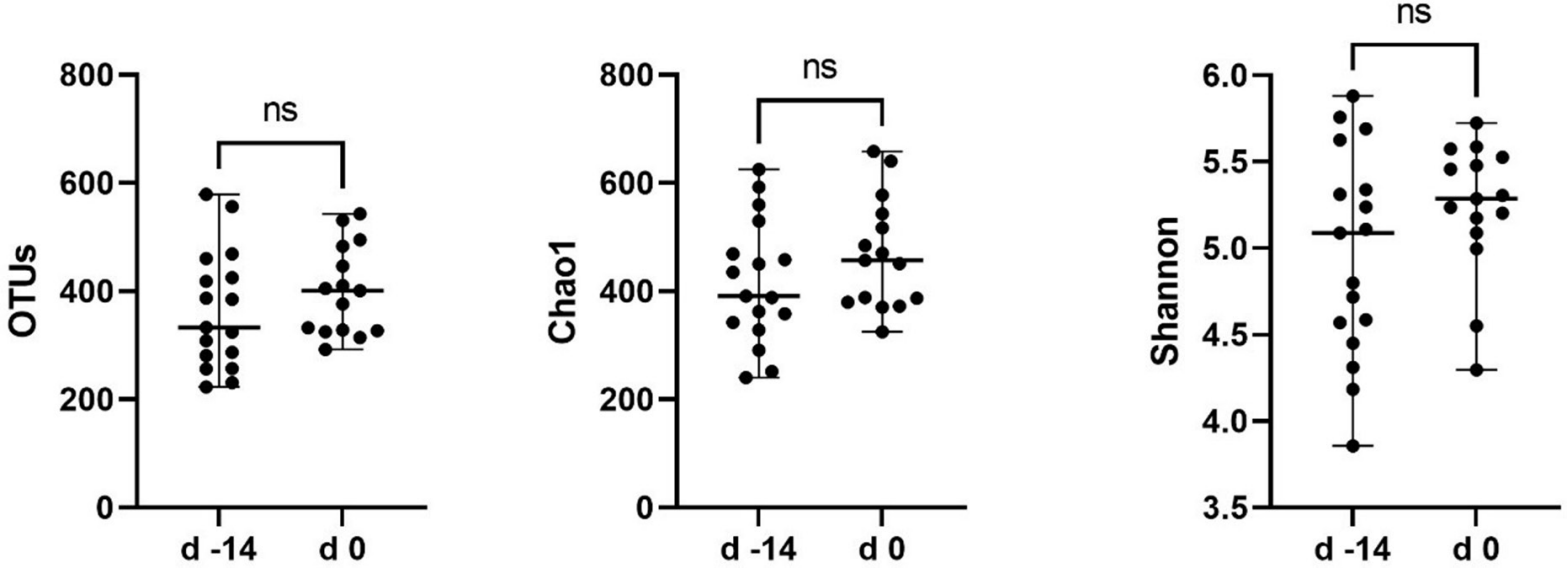
Observed OTUs, Chao1 and Shannon diversity index comparison between the baseline diet at day −14 and when all dogs were switched to a standardized diet at day 0. Wilcoxon rank comparison revealed *P*-values of 0.45 for OTUs, 0.35 for Chao1 and 0.15 for Shannon.

Prior to quality filtering, samples contained 99 501±17 945 (mean±sd) OTUs. After quality filtering and clustering of OTUs, 1964 OTUs were identified, and samples contained OTU counts of 82 104±14 848 (mean±sd). All samples were then sub-sampled to 39 442 OTU counts prior to further analysis (Tables S4 and S5). Rarefaction curves for samples plateaued, indicating that the sequencing depth was adequate. Based on these plots, observed OTU numbers reduced upon dietary change from baseline, but not significantly so (Fig. S1). Similarly, diversity indices remained unchanged (Fig. 4). Based on this, data from day 0 were considered the ‘baseline’ for all subsequent analyses.

### RS but not PSY or MTC reduce microbiota richness, but changes recovered during the washout period

**Fig. 5.**
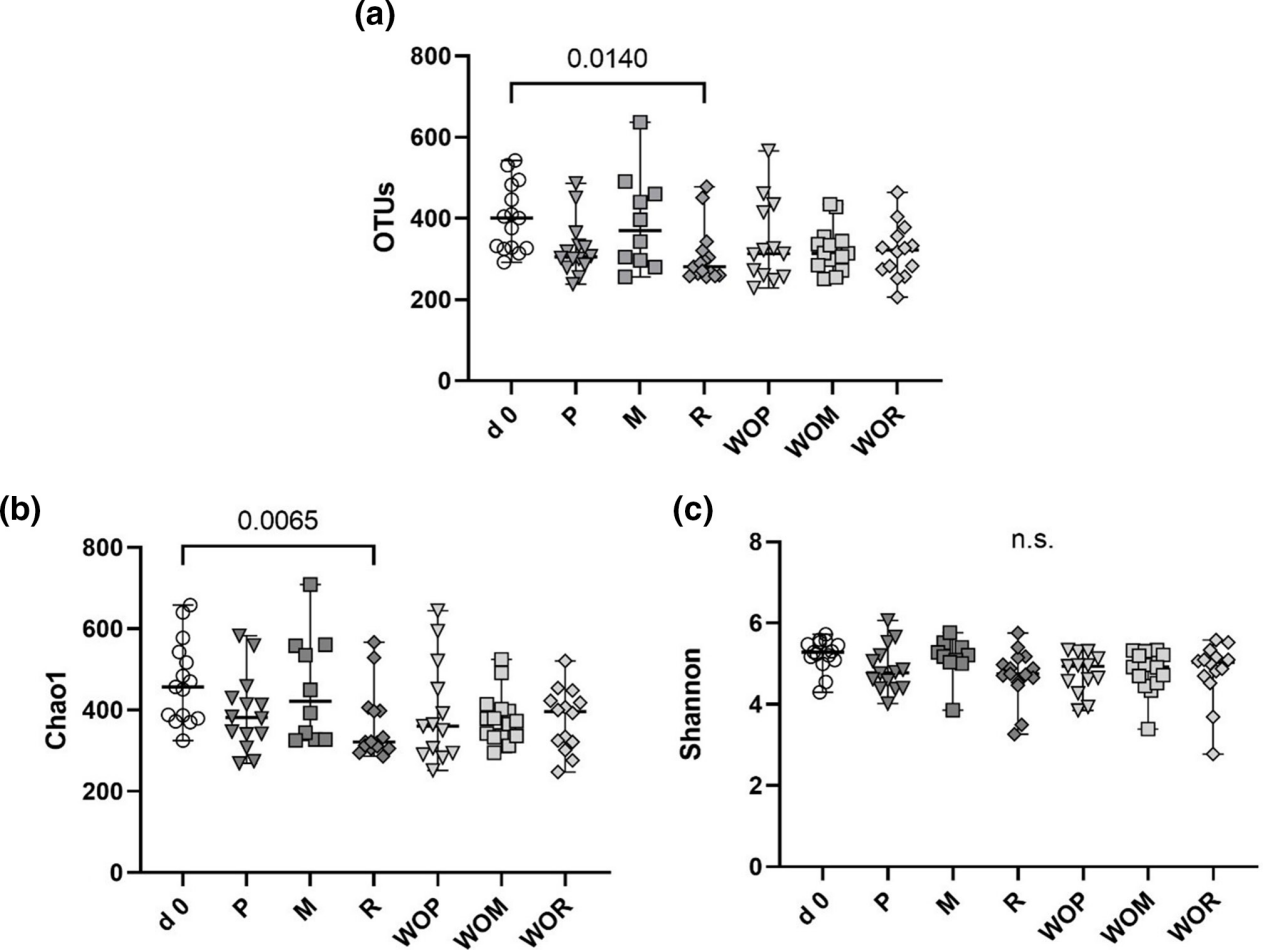
Observed OTU numbers (**a**), Chao1 (**b**) and Shannon index (**c**) across all supplementations and washouts (WO). M=methylcellulose, P=psyllium husk, R=resistant starch.

Of the different supplements, only RS resulted in significant IM changes, as evidenced by a significant reduction in observed OTUs and Chao1, but unaltered Shannon diversity (Fig. 5).

When assessing relative abundances, there was no meaningful difference on the phylum level (Fig. S2). The average composition of the most abundant families and genera were also similar across groups (Fig. S3).

**Fig. 6.**
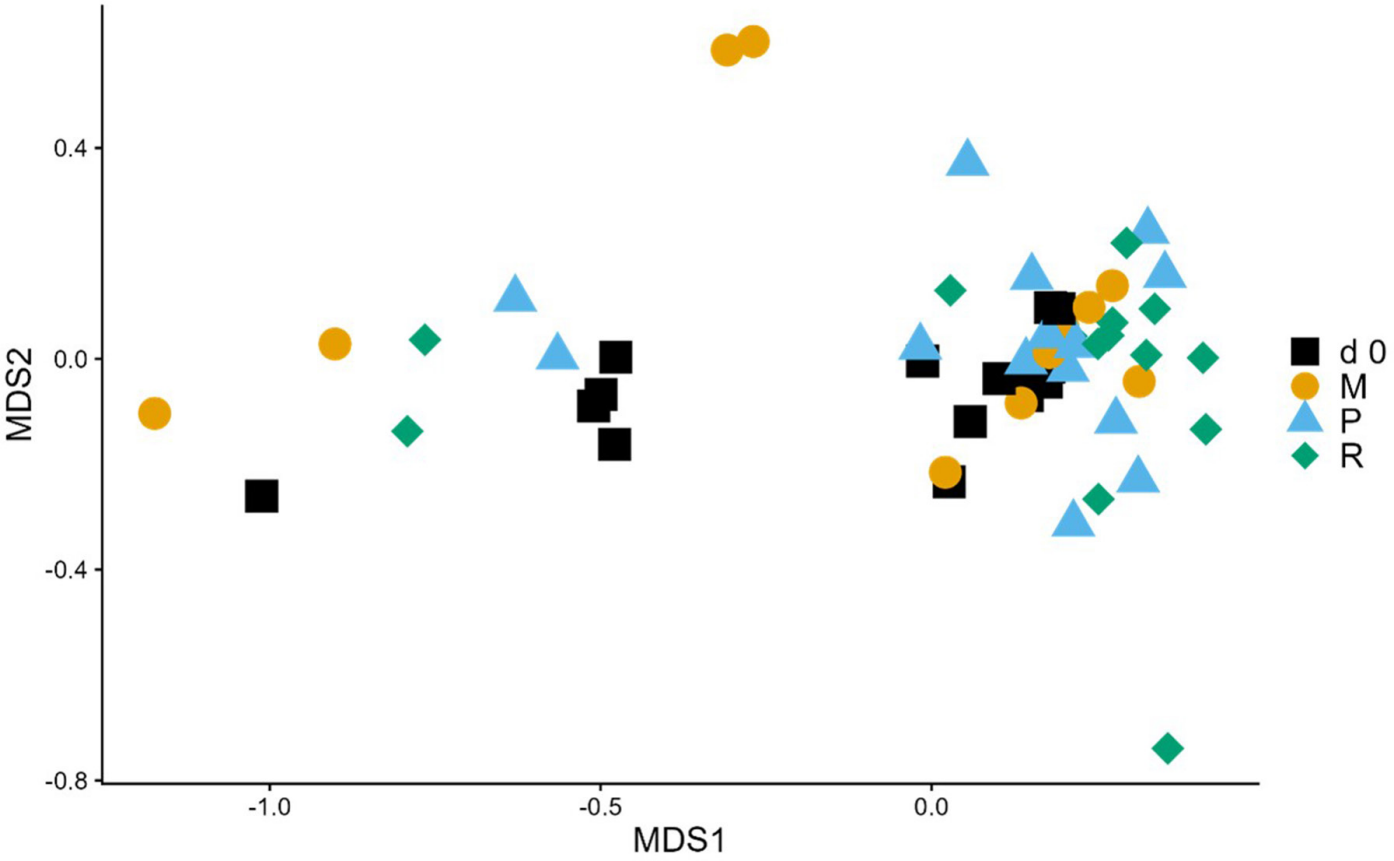
NMDS clustering samples using Bray–Curtis dissimilarity values (stress=0.14). Samples originated from baseline (d 0) or from dogs that had received a DF supplement in their diet (M=methylcellulose, P=psyllium husk, R=resistant starch). Groups did not cluster significantly by composition (PERMANOVA: *P*=0.38).

Baseline and DF-treated samples did not cluster significantly separately by their overall community composition (PERMANOVA: *P*>0.05, Fig. 6). Using Metastats to identify taxa that differed significantly between groups, 12 genera were found to be significantly differently abundant between baseline samples and PSY-treated samples (*q*<0.05, Sup_D-vs-P_metastats_genera.xls), with eight that were more abundant in baseline samples (*Deinococcus*, *Hydrogenophilus*, *Anaerotruncus*, *IS-44*, *Kocuria*, *Exiguobacterium*, *Psychroglaciecola* and *A2*) and four that were more abundant in treated samples (*Psychrobacter*, *Anaerovibrio*, *Pseudoalteromonas*, *Lysinibacillus*). However, all of these genera were low in abundance, with the most abundant per group being *Psychrobacter*, at 0.00024±0.00023 (mean±se). No genera were found to be significantly different between baseline and MTC-treated samples (Sup_D-vs-M_metastats_genera.xls).

**Fig. 7.**
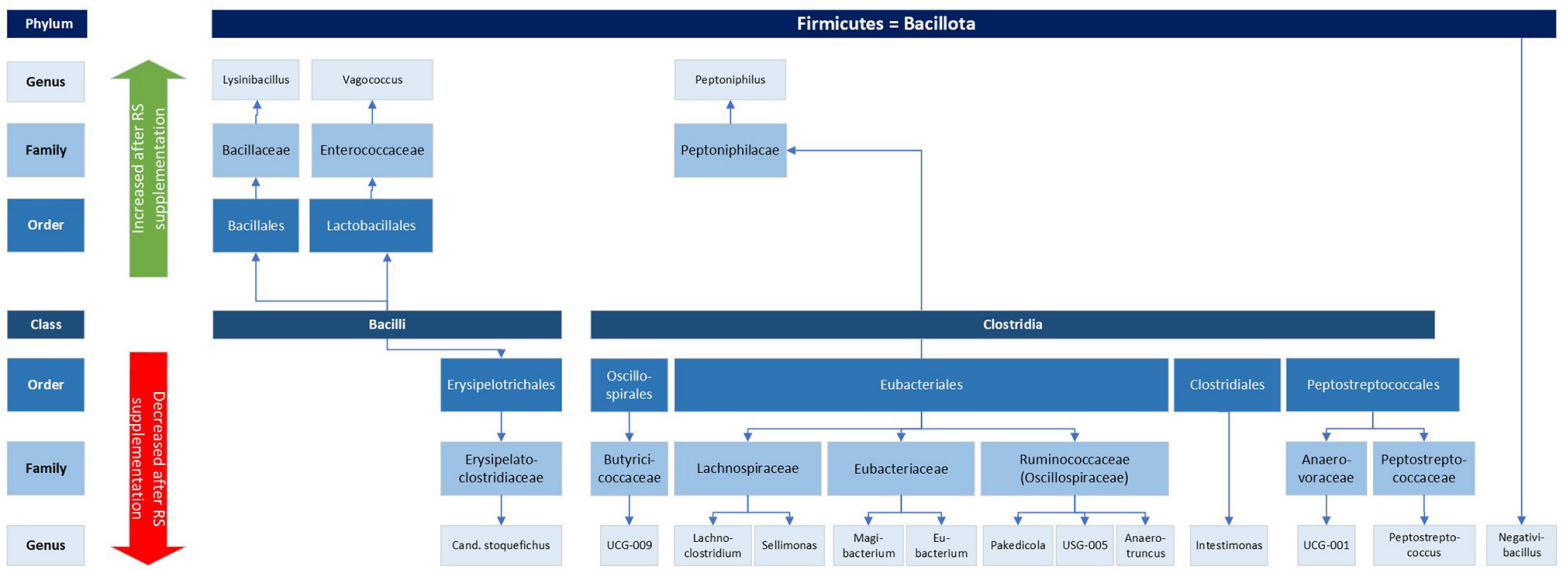
Difference in abundance of bacterial groups belonging to the phylum *Firmicutes*/*Bacillota* from samples after supplementation with resistant starch. The upper panel (indicated by green arrow) shows groups that increased after supplementation; the lower panel (indicated by red arrow) shows groups that decreased.

Thirty-seven genera were found to differ between baseline samples and samples after supplementation with RS (Sup_D-vs-R_metastats_genera.xls), with 21 being more abundant in baseline samples and 16 being more abundant in treated samples (Table 1 and Figs7  8).

**Fig. 8.**
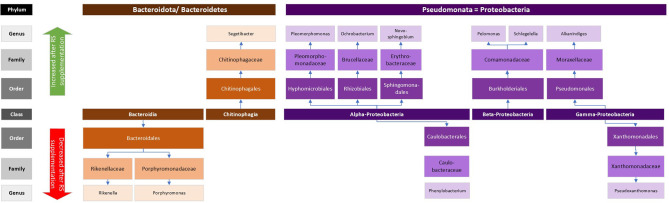
Difference in abundance of bacterial groups belonging to the phyla *Bacteroidetes*/*Bacteroidota* (left groups in shades of brown) and *Pseudomonata*/*Proteobacteria* (right groups in shades of purple) from samples after supplementation with resistant starch. The upper panel (indicated by green arrow) shows groups that increased after supplementation; the lower panel (indicated by red arrow) shows groups that decreased.

**Table 1.** Metastats for baseline samples vs samples after resistant starch (RS) supplementation, showing differentially abundant bacterial taxa only Relative abundance is reported as a value between 0 and 1, where 0 indicates the complete absence of the taxa and 1 indicates 100 % abundance.

Taxa	Mean baseline	se Baseline	Mean RS	se RS	*q* Value
*Sellimonas*	0.001674	0.000203	0.000978	0.000107	0.042502
*Negativibacillus*	0.001187	0.000185	0.000513	0.000115	0.042502
*Eubacterium_brachy_group*	0.000823	0.000175	0.000228	4.18E-05	0.042502
*UCG-005*	0.000749	9.37E-05	0.00027	4.31E-05	0.039722
*Desulfovibrio*	0.000194	8.41E-05	1.64E-05	1.02E-05	0.042502
*Intestinimonas*	0.000174	2.19E-05	8.39E-05	1.76E-05	0.042502
*A2*	0.000145	0.000145	0	0	0.024101
*Candidatus_Stoquefichus*	0.000129	2.40E-05	2.74E-05	1.32E-05	0.024101
*Rikenellaceae_RC9_gut_group*	0.000106	6.51E-05	0	0	0.042502
*Mogibacterium*	9.20E-05	3.75E-05	0	0	0.039722
*Family_XIII_UCG-001*	5.96E-05	1.72E-05	7.30E-06	5.64E-06	0.042502
*Paludicola*	3.07E-05	9.72E-06	0	0	0.042502
*Porphyromonas*	2.04E-05	1.39E-05	0	0	0.024101
*Anaerotruncus*	2.04E-05	2.04E-05	1.82E-06	1.82E-06	0.042502
*Cellulomonas*	1.70E-05	1.16E-05	0	0	0.039722
*Kapabacteriales*	1.53E-05	1.05E-05	0	0	0.042502
*Clostridium_innocuum_group*	1.53E-05	1.02E-05	0	0	0.042502
*UCG-009*	1.53E-05	1.36E-05	0	0	0.042502
*Peptostreptococcus*	1.53E-05	6.96E-06	0	0	0.042502
*Phenylobacterium*	1.53E-05	1.05E-05	0	0	0.042502
*Pseudoxanthomonas*	1.53E-05	1.53E-05	0	0	0.042502
*Blastococcus*	0	0	1.82E-05	1.12E-05	0.024101
*Microbacterium*	0	0	1.46E-05	7.91E-06	0.042502
*Brooklawnia*	0	0	1.64E-05	1.64E-05	0.032337
*Propioniciclava*	0	0	2.19E-05	2.00E-05	0.024101
*Saccharopolyspora*	0	0	2.92E-05	2.73E-05	0.024101
*Segetibacter*	0	0	0.00017	0.000153	0.024101
*Lysinibacillus*	0	0	3.65E-05	2.24E-05	0.024101
*Vagococcus*	0	0	4.20E-05	3.12E-05	0.024101
*Peptoniphilus*	0	0	1.82E-05	1.64E-05	0.024101
*TM7X*	0	0	0.000462	0.000377	0.024101
*Pleomorphomonas*	0	0	2.92E-05	2.25E-05	0.024101
*Ochrobactrum*	0	0	2.74E-05	2.74E-05	0.024101
*Novosphingobium*	0	0	1.46E-05	1.13E-05	0.042502
*Pelomonas*	0	0	6.39E-05	5.44E-05	0.024101
*Schlegelella*	0	0	4.38E-05	3.67E-05	0.024101
*Alkanindiges*	0	0	8.21E-05	8.21E-05	0.024101

**Fig. 9.**
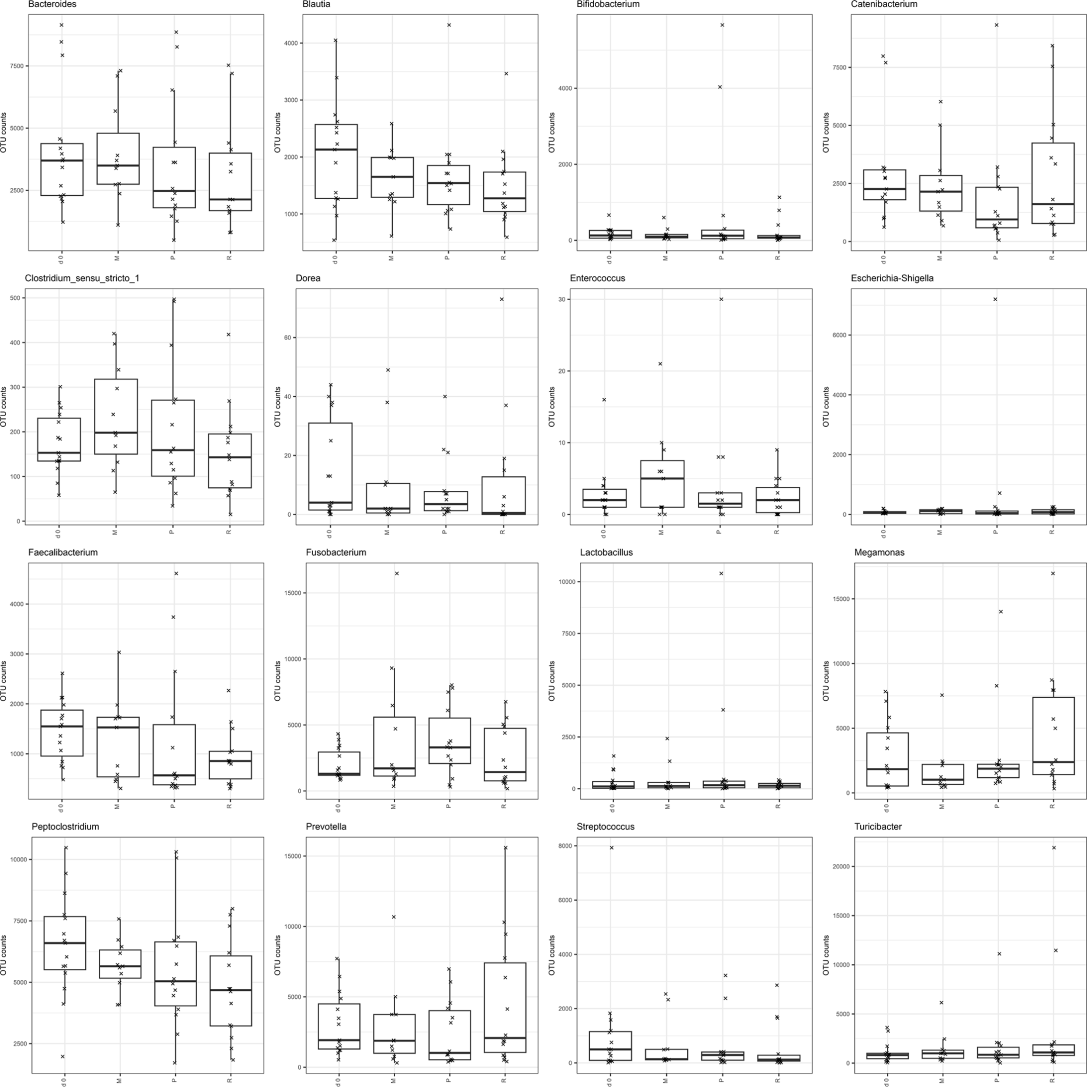
Relative abundance of selected bacterial groups at baseline (day 0) and after supplementation with different DF supplements (M=methylcellulose, P=psyllium husk, R=resistant starch).

The relative abundance of specific bacterial groups of interest (e.g. part of the ‘dysbiosis index’ [28] or associated with gut health in dogs in the literature) was also assessed. Using the Wilcoxon rank test, there was a significant decrease in *Faecalibacterium* and *Peptoclostridium* with RS supplementation (Fig. 9), but this significance was not upheld after false discovery rate control. There was no difference in the abundance of any of those selected bacteria with the other DF supplements compared to baseline.

## Discussion

This is the first study to assess and compare detailed faecal microbiota changes associated with supplementation of three types of commonly used DFs as specific supplements in dogs. While other studies have determined microbiota changes with high-fibre extruded diets (e.g. in comparison to hydrolysed or high-protein diets [29], for weight loss [30] or to modulate intestinal postbiotics [31]) or functional properties of certain types of fibre naturally occurring in raw ingredients (e.g. grains or cereals [32], miscanthus [33] or – recently – red ginseng [34]) or fibre blends [31], there is no study that compares single DFs from different broad fibre ‘categories’ in the same dogs. This seems particularly surprising for PSY, as this is one of the most commonly recommended DFs for use in intestinal disorders in dogs [35,36]. For this reason, PSY was chosen as one of the supplements in the present study. Representatives of the other DF categories were chosen based on their easy availability (e.g. household ingredients), with green banana flour as a source of RS and pure MTC (used as a gelling agent in baking, but also occasionally as a laxative [37]) as a non-digestible fibre. The latter effect of MTC was confirmed in this study, as it was the only one of the three supplements that caused a softer faecal consistency (as evidenced by significant increases in faecal scores). This did, however, not extend to any other unwanted adverse effects, as – based on the static CIBDAI indices – activity levels, appetite and other clinical parameters remained unchanged.

Overall, the effects of all three DFs on the microbiota richness and composition as assessed by 16S rRNA gene sequencing in these healthy dogs was mild, with most numerous changes found with RS supplementation, while there were no significant microbiota alterations with MTC. For PSY, while significant, differences found were in low-abundance genera, and hence these may not be biologically relevant. The most abundant change for PSY was for the genus *Psychrobacter*, which belongs to the family *Moraxellaceae*, of the order *Pseudomonadales* within the class *Gammaproteobacteria*. *Psychrobacter* is a widespread and evolutionarily successful group of bacteria and is likely to have a role as a commensal, degrading various dissolved organic carbon compounds other than sugars [38]. Some species of the genus have been isolated as cause of human infections [38], and as it is a Gram-negative bacterium carrying hypoacylated lipopolysaccharides, it induces a TLR4-mediated inflammatory response [39]. While this study does not dispute any clinical benefit seen with PSY supplementation in dogs with gastrointestinal conditions, it does not support that any benefits are derived from significant microbiota changes. However, it is possible that changes would be more evident when giving PSY to dogs with specific gastrointestinal conditions instead of healthy dogs. In addition, we did not assess microbiota function directly (e.g. measuring SCFA production). Prediction of metagenomic functions based on 16S rRNA data, for example using enrichment analysis [40] or bioinformatic tools like PICRUSt2 [41], is unlikely to be meaningful, as canine-specific databases are currently not of the desired quality, and tools are biased towards human microbiota taxa. It is possible that PSY administration was not of sufficient duration or dose to detect related microbiota changes or that changes are related to taxa or species of very low abundance, which is difficult to capture with 16S rRNA gene sequencing alone.

Supplementation with RS led to the greatest number of taxa differing in comparison to baseline samples. This may indicate that RS supplementation has a greater effect on faecal microbiota composition. This is also supported by the significant differences that were seen in richness (but not diversity indices) for this DF. Interestingly, in another study that used RS as a supplement in healthy dogs, no changes of α- or β-diversity were observed [42].

Interpretation of the type of changes in microbiota abundance with RS supplementation and their meaning is challenging, as the majority of genera are not well described with regard to their microbiological niche, main physiological function or relevance in disease in dogs. The overall observation when assessing the phylogeny is that *Firmicutes* (renamed *Bacillota*) were generally reduced in their abundance (all of the ones belonging to the class *Clostridia*, and some of the class *Bacilli*; compare Fig. 7), while *Proteobacteria* (now *Pseudomonata*) and *Bacteroidetes* (now *Bacteroidota*) were increased (compare Fig. 8). Of the 21 taxa with lower abundance after RS supplementation, eight have been associated with gut homeostasis, repair or SCFA production (*Sellimonas* [43]*, Negativibacillus* [44,45]*, Eubacterium* [46]*, UCG-005* [47]*, Intestinimonas* [48]*, Rikenella* [49]*, Anaerotruncus* [50,51]*, UCG-009* [48]). Some of these findings are in line with Beloshapka *et al*. [42], who also found *Anaerotruncus* to decrease with increased RS consumption. Furthermore, seven of the 21 taxa are considered normal commensals of the oral microbiome (*Mogibacterium* [52]), gastrointestinal tract or environmental (*Cellulomonas* and *Porphyromonas* [51]*, Paludicola* [48] *Phenylobacterium* [53]*, Pseudoxanthomonas* [54]), family *XIII UCG-001* [55], and four have been found to show differential abundance in human diseases or disease models compared to healthy controls (*Desulfovibrio* [56]*, Candidatus stoquefichus* [45] and *Clostridium innocuum* [57] are associated with colitis, while *Peptostreptococcus* was shown to increase in diabetic patients upon weight loss [58]). For two groups (*A2* and *Kapabacteriales*) no information could be found.

In contrast to the above, of the 16 taxa with increased abundance after RS treatment, six belonged to different classes of the phylum *Pseudomonadota* (previously *Proteobacteria*, see Fig. 8), five to the phylum *Actinomycetia* (*Blastococcus, Microbacterium, Saccharopolyspora, Brooklawnia* and *Propriociclava*), three to the phylum *Bacillota*/*Firmicutes*, one to the phylum *Bacteroidota* (compare Fig. 7), and one to the phylum *Saccharibacteria* (*TM7X* or *Nanosynbacter* [59]). Of these, the vast majority have been identified as being part of the environment, e.g. soil dwelling, aquatic or part of plant root microbiota [60,62]. Only two (*Vagococcus* and *Peptoniphilus* [63]) have been found as part of the human gut and reproductive tract microbiota, some with possible pathogenic potential. While it is possible that these samples had been contaminated from the environment, it seems less likely that this would only affect a specific subgroup of samples. Overall, the significance of these changes remains unclear.

None of the DFs given showed any significant effect on bacterial groups comprising the diagnostically used ‘dysbiosis index’ [28], i.e. there were no specific increases in ‘gut health’ markers such as *Faecalibacterium* or *Clostridium sensu stricto1* (which contains *Clostridium hiranonis*). Contrary to this, a significant increase in *Faecalibacterium* and *Roseburia* (both *Firmicutes*) was seen with increased RS consumption in another study [42]. These differences might be due to different types and sources of RS. For example, Beloshapka *et al*. [42] used high-amylose maize cornstarch as a source for RS.

Lastly, general limitations of next generation sequencing workflows need to be acknowledged. While we have strived to follow best practice in our methods, bias can be introduced at all methodological stages during 16S rRNA analysis [64], and this can lead to issues with reproducibility [65]. This should be taken into account when interpreting results.

## Conclusion

Overall, while MTC induced no discernible microbiota changes (but resulted in mild diarrhoea), microbiota changes seen with PSY supplementation were mild and of questionable biological relevance. In contrast, microbiota changes induced by RS supplementation did not seem favourable, albeit being based on limited available information for the observed bacterial groups. Consequently, this particular RS would not be considered a desirable prebiotic and its use cannot be recommended in dogs.

## supplementary material

10.1099/acmi.0.000774.v4 Uncited Supplementary Material 1.

10.1099/acmi.0.000774.v4 Uncited Table S1.

10.1099/acmi.0.000774.v4 Uncited Table S2.

## Abbreviations

CIBDAI: Canine IBD Activity Index
DF: dietary fibre
IM: intestinal microbiota
MTC: methylcellulose
NSP: non-starch polysaccharide
OTU: operational taxonomic unit
PFS: Purina Faecal Score
PSY: psyllium seed husk
RS: resistant starch
SCFA: short-chain fatty acid
