# Supplementary material for: Microbiota of healthy dogs demonstrate a significant decrease in richness and changes in specific bacterial groups in response to supplementation with resistant starch, but not psyllium or methylcellulose, in a randomized cross-over trial
Source: Access Microbiol. 2024 May 14;6(5):000774.v4. doi: 10.1099/acmi.0.000774.v4 (PMC11165627; doi:10.1099/acmi.0.000774.v4)
Supplement: Uncited Supplementary Material 1. [file acmi-6-00774-s001.pdf]

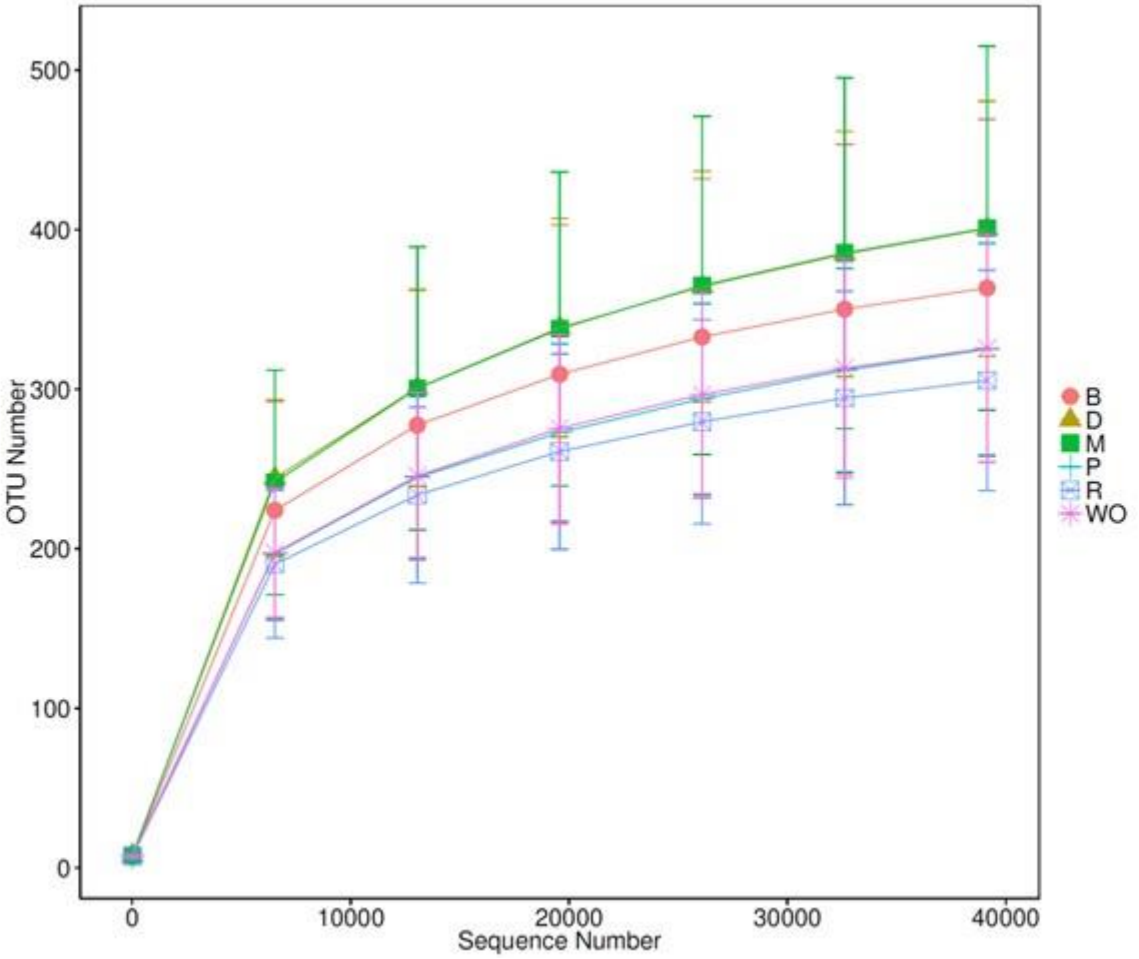


**Figure S1.** Rarefaction plot of OTU numbers from all study groups. B = baseline/ start, D = after diet change, M = methylcellulose, P = psyllium husk, R = resistant starch, WO = washout periods

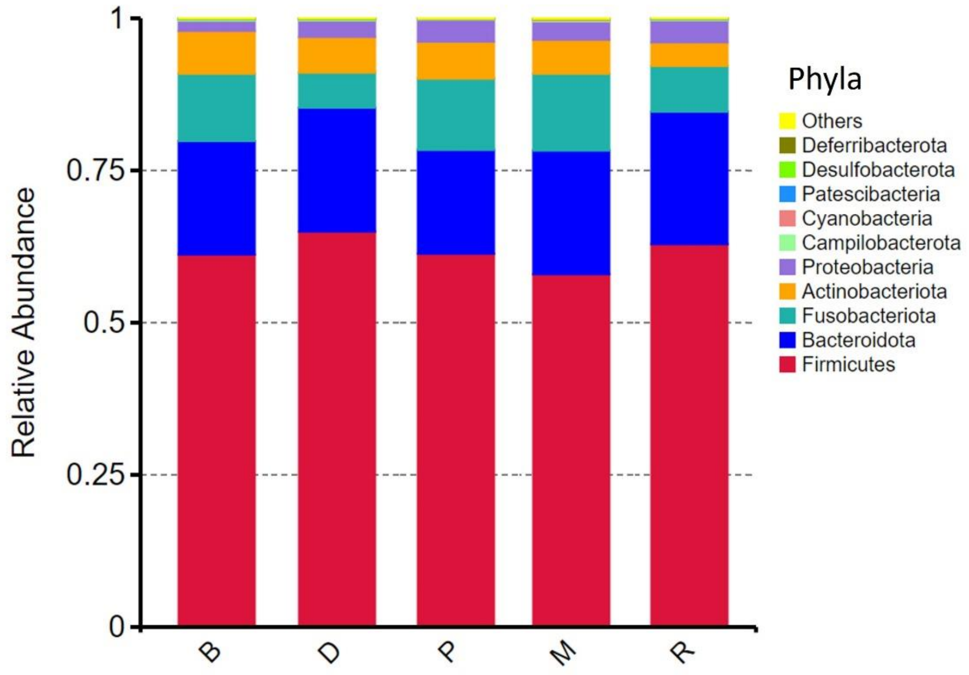


**Figure S2.** Relative abundance of phyla across different supplementation groups of the study. Showing the ten most abundant taxa, with other taxa gathered under “Others”.

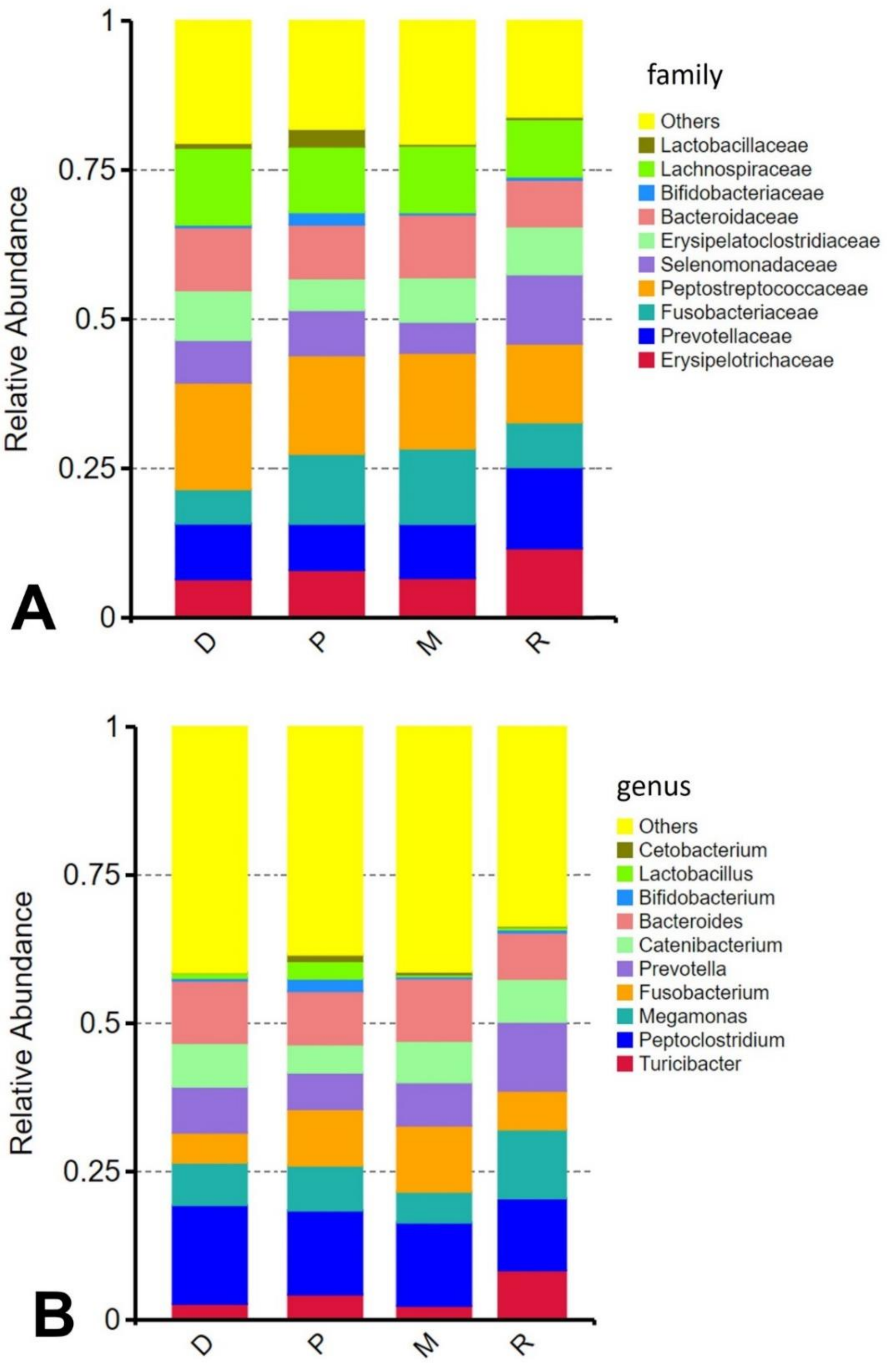


**Figure S3.** Relative abundance of bacterial families (A) and genera (B) across different supplementation groups (P, M,R) compared to samples after dietary change (D). Showing the ten most abundant taxa, with other taxa gathered under “Others”.

| Study number | Breed | weight | BCS | Gender | Age | Treatment sequence allocation |
|---|---|---|---|---|---|---|
| FIBRFLOR01 | Podengo | 10 | 6 | NF | 3 y 5 m | A |
| FIBRFLOR02 | Collie x Greyhound | 22.8 | 4 | NM | 6 y 7 m | D |
| FIBRFLOR03 | Cocker | 16.3 | 6 | NM | 5 y 6 m | C |
| FIBRFLOR04 | Dachshund | 6.1 | 5 | MN | 1 y 5 m | B |
| FIBRFLOR05 | Viszla | 28.6 | 5 | NF | 1 y 10 m | F |
| FIBRFLOR06 | Border Terrier | 9.2 | 5 | NF | 3 y 6 m | E |
| FIBRFLOR07 | English Spriner Spaniel | 15.4 | 5 | NF | 2 y 7 m | A |
| FIBRFLOR08 | Boxer | 30 | 5 | NM | 5 y 2 m | D |
| FIBRFLOR09 | Border Collie Cross | 17.5 | 5 | NF | 9 y 9 m | C |
| FIBRFLOR11 | Collie | 24 | 5 | NM | 7 y 7 m | E |
| FIBRFLOR12 | Miniature Labradoodle | 17.3 | 6 | NM | 4 y0 m | F |
| FIBRFLOR14 | Rottweiler | 36.6 | 5 | NM | 6 y 11 m | B |
| FIBRFLOR16 | Pug | 8.7 | 6 | NM | 6 y 6 m | C |
| FIBRFLOR17 | Pug | 9.4 | 7 | M | 8 y 9 m | A |
| FIBRFLOR18 | Staffordshire Terrier - cross | 23.15 | 6 | NF | 5 y | E |
| FIBRFLOR21 | Dalmatian | 20 | 5 | NF | 6 y 1 m | F |
| FIBRFLOR22 | Cross breed | 22 | 5 | F | 1 y 7 m | C |

**Table S1.** Signalement of included dogs and their treatment sequence allocation. BCS is out of 9; treatment sequence explanation is provided in figure 1; N = neutered, F = female, M = male

| Block | n | First | Second | Third |
|---|---|---|---|---|
| A | 3 | PSY | RS | MTC |
| B | 2 | PSY | MTC | RS |
| C | 4 | RS | PSY | MTC |
| D | 2 | MTC | PSY | RS |
| E | 3 | MTC | RS | PSY |
| F | 3 | RS | MTC | PSY |

**Table S2.** Randomization of dogs (n) for the study. Each were randomized into a block (A-F), which were designed to allow an even distribution of supplements as first, second and third treatment in the cross-over trial. MTC = methylcellulose, PSY = psyllium husk, RS = resistant starch.

| Samples | Day -14 | Day 0 | PSY | MTC | RS |
|---|---|---|---|---|---|
| All | 17 | 15 | 17 | 15 | 17 |
| First | n.a. | n.a. | 5 | 5 | 7 |
| Second | n.a. | n.a. | 6 | 5 | 6 |
| Third | n.a. | n.a. | 6 | 7 | 4 |

**Table S3**. Availability of samples from 17 dogs completing the study, indicating samples from which DF supplementation were available for analysis. n.a. = not applicable. PSY = psyllium husk, MTC = methylcellulose, RS = resistant starch.
